# Carvedilol triggers ferroptosis in hepatic stellate cells via the ATF4/SAT1 axis promoting spermidine depletion to ameliorate liver fibrosis

**DOI:** 10.1038/s41419-026-08898-5

**Published:** 2026-05-22

**Authors:** Yifu Xia, Sining Wang, Junyuan Zhu, Yuwen Chen, Xu Liu, Chunqing Zhang

**Affiliations:** 1 Department of Gastroenterology, Shandong Provincial Hospital Affiliated to Shandong First Medical University, Jinan, China; 2 Department of Critical Care Medicine, Shandong Provincial Hospital Affiliated to Shandong First Medical University, Jinan, China; 3 Department of Infectious Diseases and Hepatology, The Second Hospital of Shandong University, Jinan, China

**Keywords:** Cell death, Cell signalling

## Abstract

Liver fibrosis is a major pathological consequence of chronic liver injury, and carvedilol, a drug effective in reducing portal hypertension, has been shown to exert anti-fibrotic effects in recent studies, yet the precise molecular mechanism underlying this activity remains elusive. This study aimed to investigate the core scientific question of whether carvedilol alleviates liver fibrosis by inducing ferroptosis in hepatic stellate cells (HSCs) and to clarify the corresponding regulatory mechanisms. Using a CCl_4_-induced mouse liver fibrosis model and human (LX-2)/rat (HSC-T6) HSC lines treated with carvedilol, mechanistic analyses were conducted via Western blot, quantitative real-time PCR (RT-qPCR), RNA sequencing (RNA-seq), chromatin immunoprecipitation (ChIP), as well as functional assays involving genetic manipulation and pharmacological inhibitors. Carvedilol significantly attenuated HSCs activation and ameliorated liver fibrosis in mice, and it induced ferroptosis in HSCs—characterized by mitochondrial shrinkage, lipid peroxidation, and iron accumulation—an effect that was abrogated by the ferroptosis inhibitor ferrostatin-1. Mechanistically, carvedilol triggered endoplasmic reticulum (ER) stress to activate the PERK/eIF2α/ATF4 signaling axis, and ATF4 transcriptionally upregulated spermidine/spermine N1-acetyltransferase 1 (SAT1). Ectopic overexpression of SAT1 alone was sufficient to induce ferroptosis and suppress HSCs activation, whereas SAT1 knockdown completely abolished the aforementioned effects of carvedilol. Importantly, SAT1 catalyzed spermidine depletion, which led to the post-transcriptional downregulation of GPX4 and xCT proteins, and exogenous spermidine supplementation effectively rescued SAT1-overexpression-induced ferroptosis in HSCs. Collectively, our findings demonstrate that carvedilol ameliorates liver fibrosis by inducing HSCs ferroptosis via the ER stress/ATF4/SAT1/spermidine depletion axis. This study identifies a novel regulatory role of spermidine metabolism in HSCs ferroptosis and liver fibrogenesis, and further establishes spermidine metabolism as a potential and specific therapeutic target for fibrotic liver diseases.

## Introduction

Liver fibrosis is a common pathological outcome of chronic liver injury, characterized by excessive deposition of extracellular matrix (ECM) [1]. Hepatic stellate cells (HSCs) are the primary effector cells in this process. Upon activation, HSCs transdifferentiate into myofibroblasts, proliferate excessively, and secrete large amounts of ECM proteins such as collagen, leading to architectural distortion and impaired liver function [2, 3]. Consequently, targeting HSCs activation and survival has emerged as a central strategy for anti-fibrotic therapy [4].

Ferroptosis, an iron-dependent form of regulated cell death driven by lethal lipid peroxidation, has recently gained attention as a promising therapeutic target for various diseases, including liver fibrosis [5, 6]. This distinct form of cell death is characterized by glutathione (GSH) depletion, inactivation of glutathione peroxidase 4 (GPX4), and accumulation of reactive oxygen species (ROS) and lipid peroxides [7, 8]. Notably, induction of ferroptosis in activated HSCs has been demonstrated to effectively alleviate liver fibrosis, highlighting its significant potential as a novel anti-fibrotic strategy [9, 10]. For instance, artesunate promotes HSCs ferroptosis via the ROCK1/ATF3 pathway, leading to the inhibition of the antioxidant system and amelioration of fibrosis [11]. Doxofylline (DOX) exerts anti-fibrotic effects by regulating the ferroptosis signaling pathway, which can be reversed by the ferroptosis inhibitor deferoxamine [12].

Carvedilol is a non-selective β-adrenergic blocker with additional α1-adrenergic blocking activity, widely used as a first-line treatment for portal hypertension in cirrhotic patients [13]. Although it is known to reduce intrahepatic vascular resistance more effectively than conventional NSBBs, the exact mechanisms remain poorly defined. Beyond its hemodynamic effects, growing evidence suggests that carvedilol possesses direct anti-fibrotic properties [14, 15]. However, the precise molecular mechanisms underlying its anti-fibrotic effects, particularly whether it involves the induction of novel cell death pathways like ferroptosis in HSCs, remain largely unclear and require further investigation.

Based on the aforementioned evidence, we hypothesized that carvedilol may alleviate liver fibrosis by selectively inducing ferroptosis in activated HSCs. This study aims to systematically investigate the role and mechanisms of carvedilol-triggered ferroptosis in HSCs. Our findings provide new insights into the anti-fibrotic mechanisms and identify spermidine metabolism as a potential target for treating fibrotic liver diseases.

## Results

### Carvedilol suppresses HSCs activation and ameliorates CCl_4_-induced liver fibrosis in mice

**Fig. 1:**
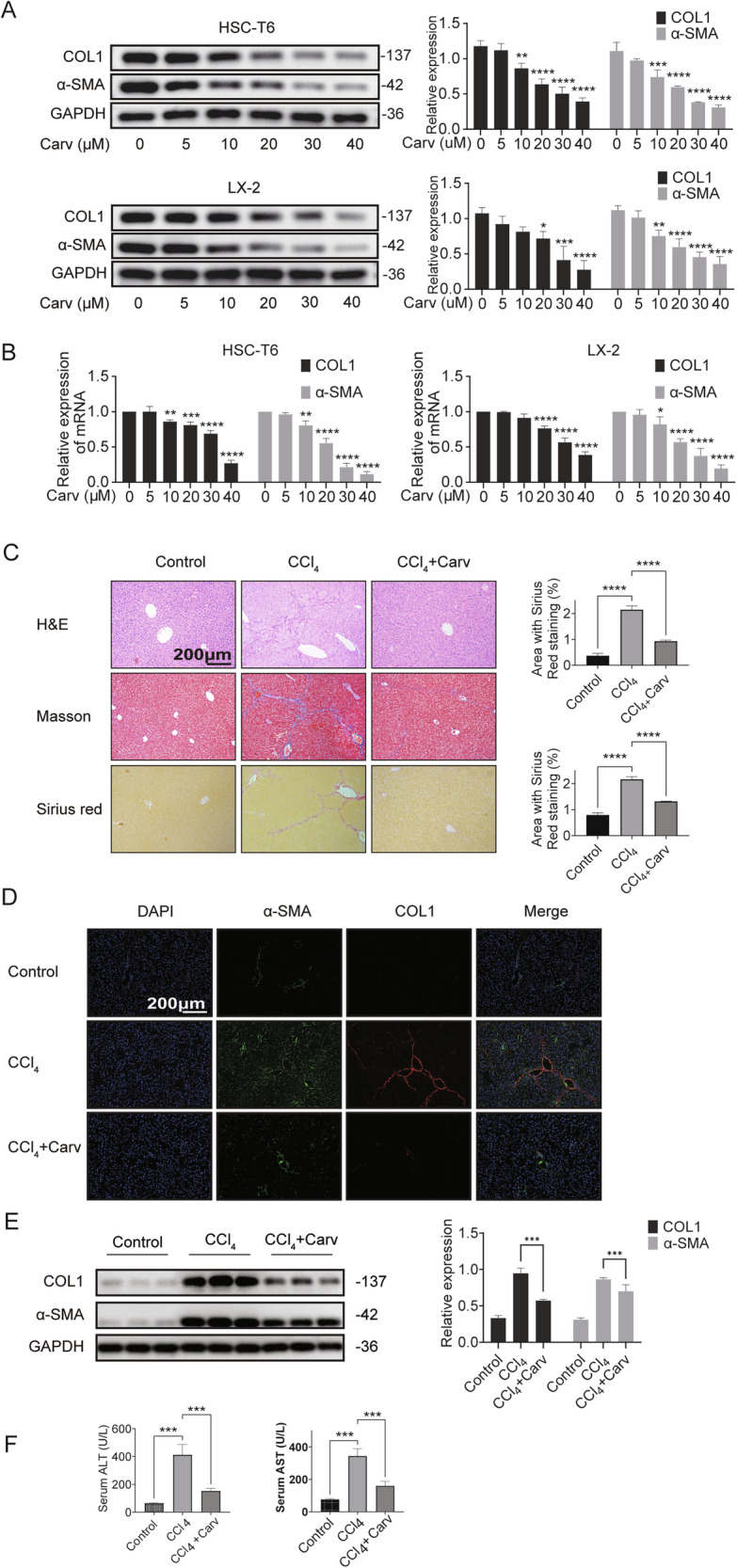
Carvedilol inhibits HSCs activation and alleviates liver fibrosis in mice. Western blot analysis of collagen I and α-SMA protein levels in HSC-T6 (**A**) and LX-2 (**B**) cells treated with the indicated concentrations of carvedilol for 24 h. **C** Representative images of H&E, Masson, and Sirius red staining of liver sections from CCl_4_-induced fibrotic mice treated with or without carvedilol (10 mg/kg). Scale bars, 200 μm. **D** Immunofluorescence staining of α-SMA (green) and collagen I (red) in liver tissues from the experimental mice. Nuclei were counterstained with DAPI (blue). Scale bars, 200 μm. **E** The expression levels of collagen I and α-SMA were detected by Western blot in CCl_4_-induced mouse liver fibrosis. **F** The levels of alanine aminotransferase (ALT) and aspartate aminotransferase (AST) in the different treatment groups. (*p < 0.05, **p < 0.01, ***p < 0.001, ****p < 0.0001). Data are representative of three independent experiments; *n* = 5 in every group.

To investigate the anti-fibrotic potential of carvedilol, we first treated activated LX-2 and HSC-T6 cells with increasing concentrations of the drug. Western blot analysis revealed that carvedilol dose-dependently reduced the protein levels of the classic activation markers α-smooth muscle actin (α-SMA) and collagen type I (Fig. 1A, B).

We next evaluated the therapeutic potential of carvedilol in vivo using a CCl_4_-induced mouse model of liver fibrosis (Supplementary Fig. 1). Histological examination of liver sections via H&E, Masson, and Sirius red staining demonstrated that carvedilol administration significantly attenuated collagen deposition and architectural distortion (Fig. 1C). This improvement was further confirmed by a marked reduction in α-SMA and collagen I expression, as visualized by immunofluorescence staining (Fig. 1D). The levels of alanine aminotransferase (ALT) and aspartate aminotransferase (AST) also demonstrated the fibrosis improvement effect of carvedilol (Fig. 1E). These in vivo findings are consistent with our cellular data and with our previous research [14, 16, 17], confirming the potent anti-fibrotic activity of carvedilol.

### Carvedilol induces ferroptosis in HSCs

**Fig. 2:**
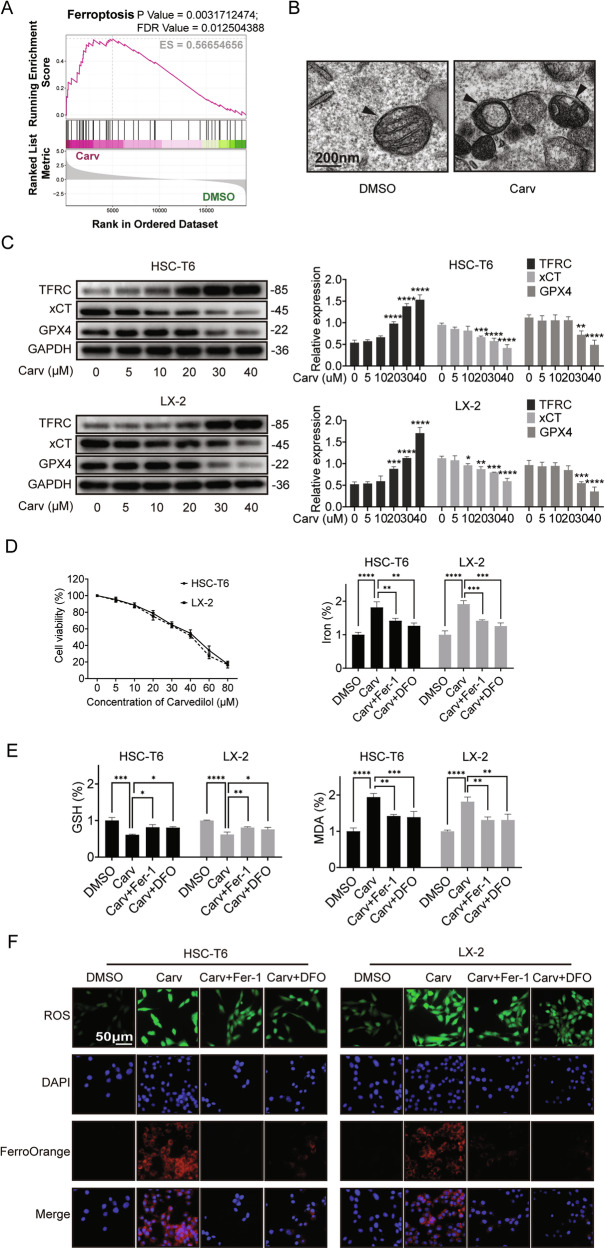
Carvedilol induces ferroptosis in HSCs. **A** GSEA plot showing enrichment of ferroptosis-related genes in HSCs treated with 30 μM carvedilol. **B** Representative TEM images of mitochondria in control and carvedilol-treated (30 μM) HSCs. Black arrows indicate shrunken mitochondria with increased membrane density and loss of cristae, characteristic of ferroptosis. Scale bars, 200 nm. **C** Western blot analysis of TFRC, xCT, and GPX4 in HSCs treated with carvedilol at the indicated concentrations. **D** Cell viability of HSCs measured by CCK-8 assay after carvedilol treatment. **E** Combined treatment with Fer-1 (1 μM) and DFO (100 μM) effectively alleviated the significant change of Fe²⁺, GSH, and MDA in HSCs induced by carvedilol (30 μM). **F** Representative fluorescence images of intracellular ROS (green) and Fe²⁺ (red) levels in HSCs following treatment with 30 μM carvedilol with or without Fer-1 (1 μM) and DFO (100 μM). Nuclei were counterstained with DAPI (blue). Scale bars, 50 μm. (*p < 0.05, **p < 0.01, ***p < 0.001, ****p < 0.0001). Data are representative of three independent experiments.

To elucidate the mechanism underlying carvedilol-induced HSCs suppression, we performed RNA sequencing. Gene Set Enrichment Analysis (GSEA) of carvedilol-treated HSCs indicated a significant enrichment of ferroptosis-related pathways (Fig. 2A), and Kyoto Encyclopedia of Genes and Genomes (KEGG) pathway enrichment analysis was conducted to identify and rank the cellular pathways modulated by carvedilol, which further verified that carvedilol mainly triggered the elevation of ferroptosis, but not other forms of cell death (Supplementary Fig. 2). Transmission electron microscopy (TEM) of treated HSC-T6 cells further confirmed the execution of ferroptosis, revealing characteristic ultrastructural features, including shrunken mitochondria with increased membrane density and loss of cristae (Fig. 2B). Consistent with these findings, carvedilol treatment led to a dose-dependent upregulation of the iron importer TFRC and downregulation of the core ferroptosis defenders xCT and GPX4 at the protein level (Fig. 2C). In vivo experiments further confirmed that carvedilol induced significant alterations in ferroptosis-related markers in the livers of mice, which were consistent with the findings from in vitro cellular experiments (Supplementary Fig. 3). A concentration of 30 μM was selected for all subsequent in vitro experiments [18, 19], as this dose exerted a robust inhibitory effect on the viability of HSCs (Fig. 2D), while showing no significant cytotoxicity toward AML12 hepatic parenchymal cells (Supplementary Fig. 4).

The involvement of ferroptosis was functionally confirmed using the specific inhibitor ferrostatin-1 (Fer-1) and deferoxamine (DFO). Co-treatment with Fer-1 and DFO effectively rescued carvedilol-induced accumulation of intracellular Fe²⁺ and malondialdehyde (MDA), and prevented the depletion of glutathione (GSH) in HSCs (Fig. 2E). The elevation in intracellular Fe²⁺ and ROS was directly visualized by specific fluorescent staining (Fig. 2F). Collectively, these data establish that ferroptosis is the primary form of cell death induced by carvedilol in HSCs.

### SAT1 is a key mediator upregulated by carvedilol in HSCs

**Fig. 3:**
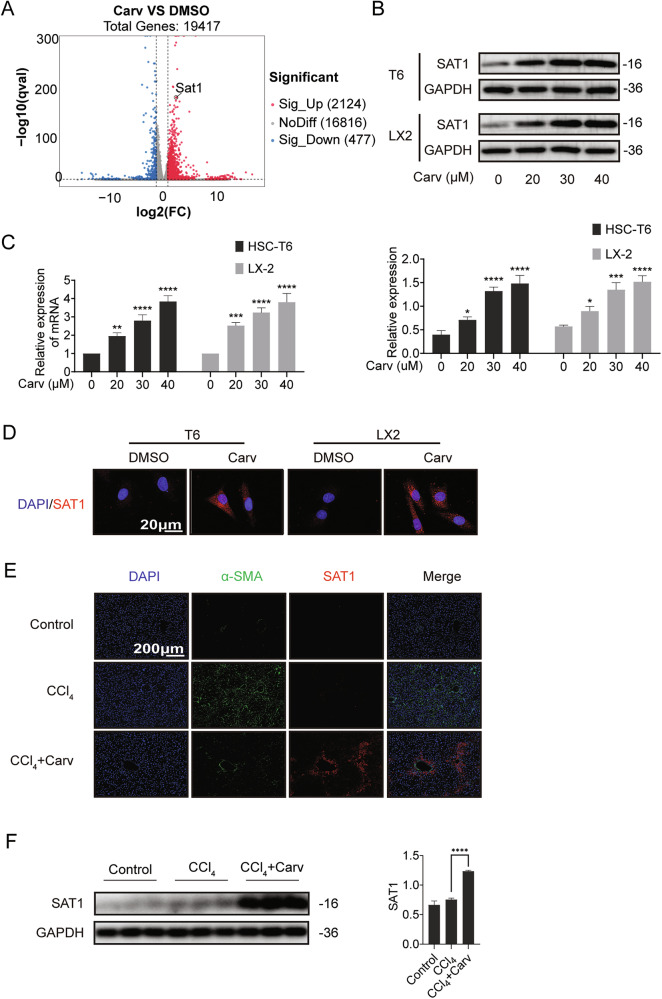
Carvedilol upregulates SAT1 expression in HSCs. **A** Heatmap from RNA-seq data showing upregulated ferroptosis-related genes, including SAT1, in HSC-T6 after carvedilol (30 μM) treatment. Western blot (**B**) and RT-qPCR (**C**) analysis of SAT1 expression in HSCs treated with the indicated concentrations of carvedilol. **D** Immunofluorescence staining of SAT1 (red) in HSCs treated with carvedilol (30 μM). Nuclei were counterstained with DAPI (blue). Scale bars, 20 μM. **E** Immunofluorescence staining of α-SMA (green) and SAT1 (red) in liver tissues from CCl_4_-induced fibrotic mice treated with or without carvedilol (10 mg/kg). Nuclei were counterstained with DAPI (blue). Scale bars, 200 μm. **F** The expression levels of SAT1 were detected by Western blot in CCl_4_-induced mouse liver fibrosis. (*p < 0.05, **p < 0.01, ***p < 0.001, ****p < 0.0001). Data are representative of three independent experiments; *n* = 5 in every group.

Analysis of our RNA-seq data for ferroptosis-related genes identified SAT1 as one of the most significantly upregulated transcripts (Fig. 3A). This induction was verified at both the protein and mRNA levels, with carvedilol treatment eliciting a potent, dose-dependent increase in SAT1 expression in HSC-T6 and LX-2 cells (Fig. 3B, C). Immunofluorescence staining robustly confirmed the increase in SAT1 protein within carvedilol-treated HSCs in vitro (Fig. 3D). Importantly, this effect was also observed in vivo, as evidenced by heightened SAT1 immunofluorescence and Western blot analysis in the livers of carvedilol-treated fibrotic mice (Fig. 3E, F).

### SAT1 mediates carvedilol-induced ferroptosis and inhibition of HSCs activation

**Fig. 4:**
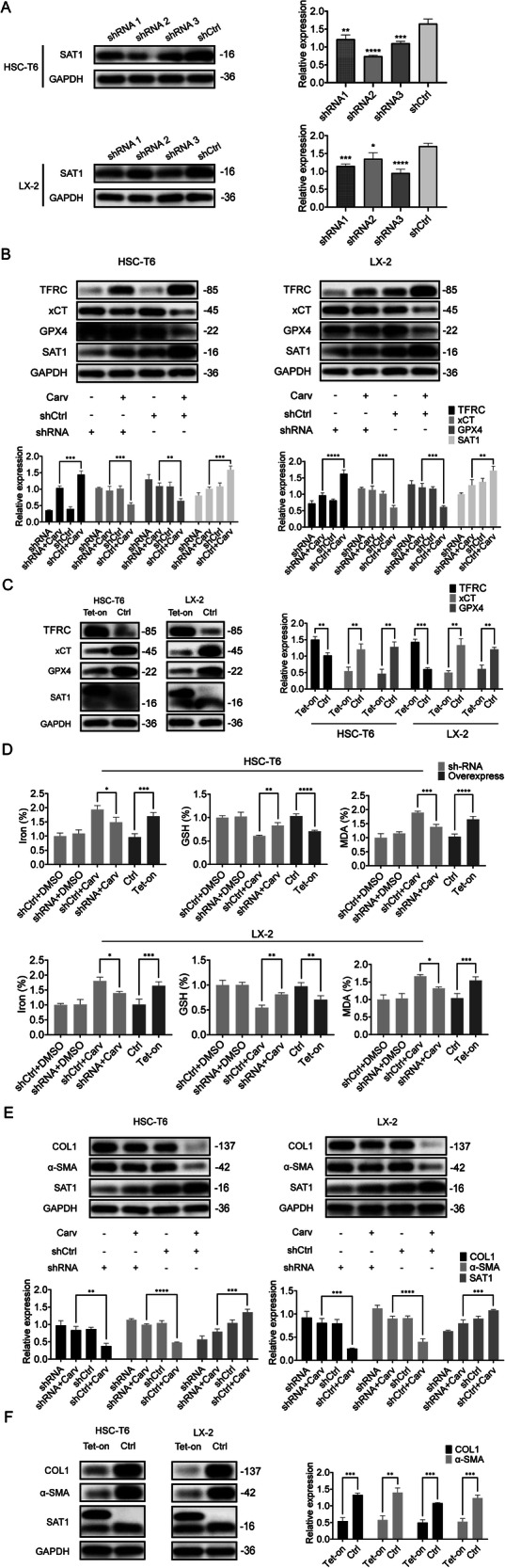
SAT1 mediates carvedilol-induced ferroptosis and suppression of HSCs activation. **A** Western blot confirming the efficiency of SAT1 knockdown using lentiviral shRNA. **B** Western blot analysis of TFRC, xCT, and GPX4 in SAT1-knockdown HSCs treated with carvedilol (30 μM). **C** Western blot analysis of TFRC, xCT, and GPX4 in HSCs with tetracycline-induced SAT1 overexpression. Western blot analysis of α-SMA and collagen I in SAT1-knockdown (**D**) or SAT1-overexpressing (**E**) HSCs with or without carvedilol (30 μM) treatment. **F** Measurements of Fe²⁺, GSH, and MDA levels in the indicated groups. (*p < 0.05, **p < 0.01, ***p < 0.001, ****p < 0.0001). Data are representative of three independent experiments.

To establish the functional role of SAT1, we generated stable SAT1-knockdown and SAT1-overexpressing HSC lines using lentiviral vectors (Fig. 4A). Knockdown of SAT1 markedly attenuated the carvedilol-induced ferroptosis marker proteins, as shown by suppressed TFRC induction and restored xCT and GPX4 protein levels (Fig. 4B). Conversely, tetracycline-induced SAT1 overexpression alone was sufficient to recapitulate the ferroptosis phenotype (Fig. 4C). Consistent with these findings, the carvedilol-induced dysregulation of Fe²⁺, GSH, and MDA was blunted by SAT1 knockdown, while SAT1 overexpression mimicked these effects (Fig. 4D).

Crucially, modulating SAT1 expression directly impacted HSCs activation. SAT1 knockdown reduced the suppressive effect of carvedilol on the expression of α-SMA and collagen I (Fig. 4E), while SAT1 overexpression alone significantly reduced the protein levels of these activation markers (Fig. 4F), demonstrating that SAT1 is not only necessary but also sufficient to drive the loss of the activated myofibroblast phenotype, likely through inducing ferroptosis.

We also examined whether the levels of autophagy or apoptosis were altered in HSCs. Western blot analysis demonstrated that SAT1 overexpression did not induce significant changes in the expression levels of autophagic (P62, LC3B) and apoptotic (Cleaved Caspase-3, Bax, Bcl-2) markers in HSCs (Supplementary Fig. 5), which further confirmed that the carvedilol-induced upregulation of SAT1 exerts its regulatory effects through ferroptosis rather than other forms of cell death.

### Carvedilol upregulates SAT1 through PERK/eIF2α/ATF4 pathway

**Fig. 5:**
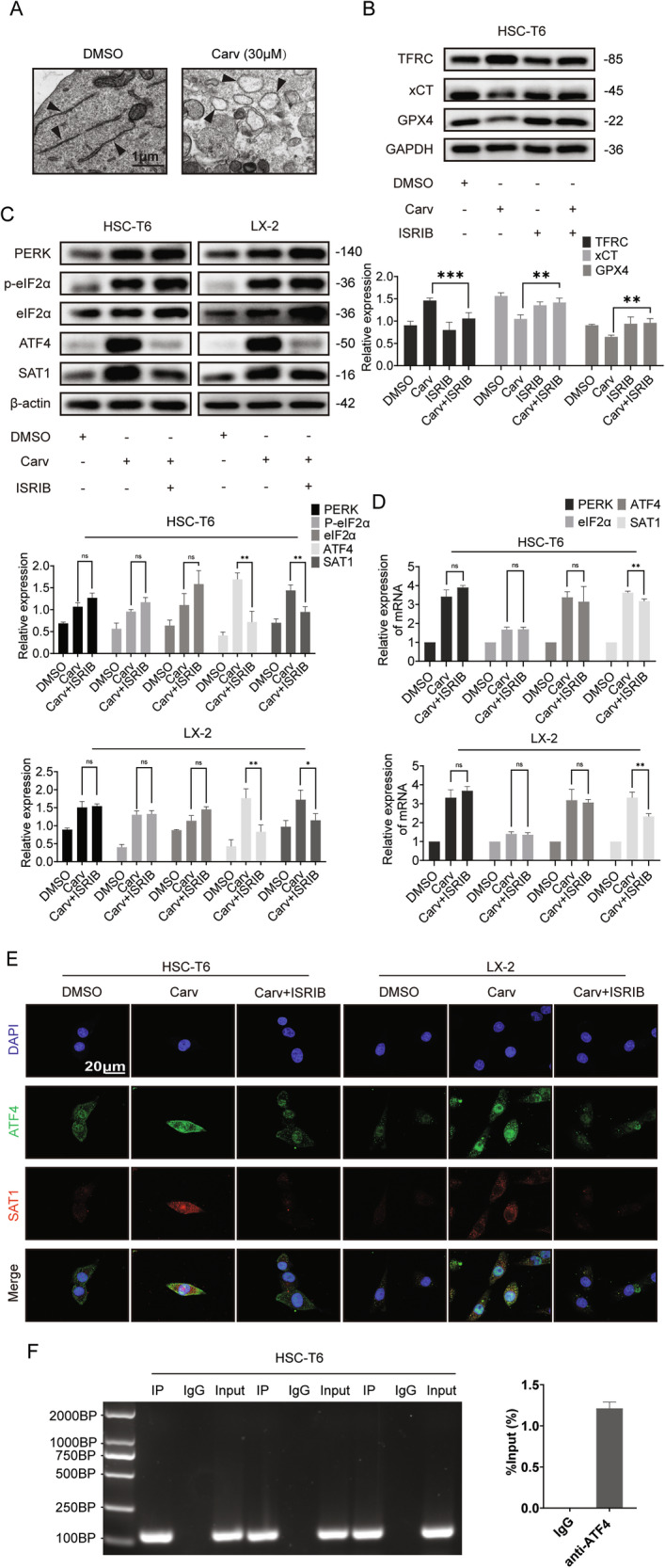
Carvedilol upregulates SAT1 through the PERK/eIF2α/ATF4 pathway. **A** TEM images showing dilated endoplasmic reticulum (black arrows) in HSCs after carvedilol (30 μM) treatment. Scale bars, 1 μm. **B** The expression levels of TFRC, xCT, and GPX4 were detected by Western blot in HSCs treated with carvedilol (30 μM) with or without the ER stress inhibitor ISRIB (200 nM). Western blot (**C**) and RT-qPCR (**D**) analysis of PERK, p-eIF2α, eIF2α, ATF4, and SAT1 in HSCs treated with carvedilol (30 μM) with or without the ER stress inhibitor ISRIB (200 nM). **E** Immunofluorescence staining of ATF4 (green) and SAT1 (red) in HSCs under the indicated treatments. Nuclei were counterstained with DAPI (blue). Scale bars, 20 μM. **F** ChIP-qPCR analysis showing the enrichment of ATF4 on the SAT1 promoter region. (*p < 0.05, **p < 0.01, ***p < 0.001, ****p < 0.0001). Data are representative of three independent experiments.

Our TEM observations revealed dilated ER in carvedilol-treated HSCs (Fig. 5A), and pathway analysis of RNA-seq data suggested ER stress involvement (Supplementary Fig. 6), collectively indicating that ER stress might underlie SAT1 induction. Western blot analysis revealed that the ER stress inhibitor ISRIB significantly downregulated TFRC expression and concurrently upregulated GPX4 and xCT expressions in carvedilol-treated HSCs, which confirmed the involvement of ER stress in the regulatory effects of carvedilol (Fig. 5B). Then, we investigated the PERK/eIF2α/ATF4 pathway, a critical branch of the unfolded protein response activated during ER stress [20]. Carvedilol treatment increased the phosphorylation of eIF2α and the protein levels of ATF4, which was accompanied by a concurrent rise in SAT1 (Fig. 5B). Importantly, ISRIB effectively suppressed the carvedilol-mediated upregulation of SAT1 (Fig. 5B, C). Immunofluorescence analysis corroborated these findings (Fig. 5D). To determine if ATF4 directly regulates *SAT1* transcription, we performed ChIP-qPCR, which confirmed significant enrichment of ATF4 on the *SAT1* promoter (Fig. 5E).

We also examined other canonical ATF4-associated signaling pathways, including cellular autophagy and apoptosis. Experimental results demonstrated that compared with the carvedilol group, the expression levels of autophagic markers (P62, LC3B) and apoptotic markers (Cleaved Caspase-3, Bax, Bcl-2) exhibited no significant alterations after ISRIB administration. Meanwhile, CCK-8 assay results showed that the ferroptosis inhibitors Fer-1 and DFO both markedly attenuated the carvedilol-induced decrease in HSCs viability, whereas rapamycin and Z-DEVD-FMK exerted no obvious rescue effects on the viability of carvedilol-stimulated HSCs (Supplementary Fig. 7). These findings indicated that ferroptosis is the primary mechanism underlying the ER stress-mediated reduction in HSCs viability induced by carvedilol.

### SAT1 triggers ferroptosis by depleting spermidine

**Fig. 6:**
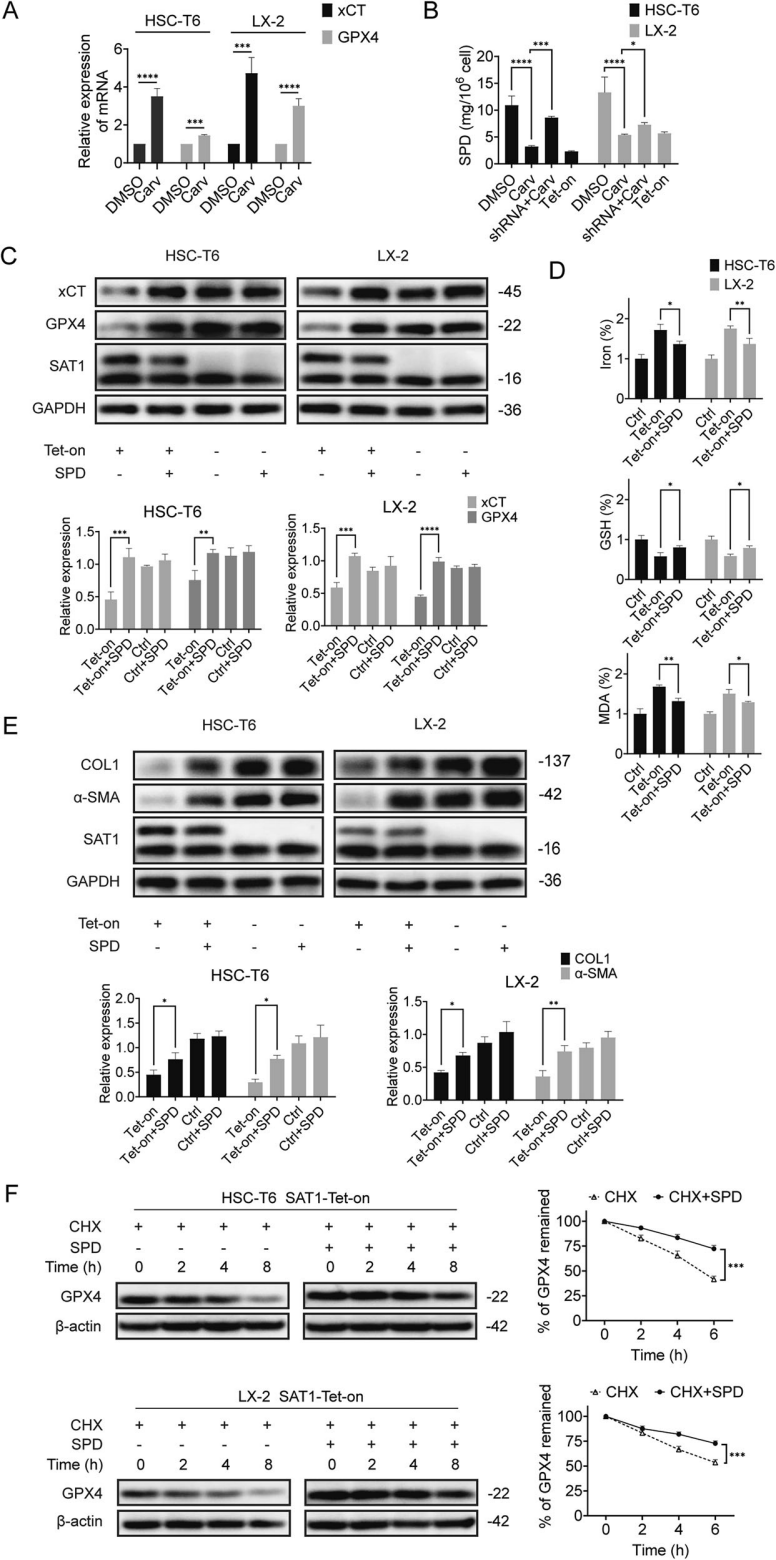
Exogenous spermidine rescues SAT1 overexpression-induced ferroptosis and HSCs activation. **A** RT-qPCR analysis of xCT and GPX4 mRNA levels in HSCs treated with carvedilol. **B** Intracellular spermidine levels measured by HPLC in HSCs. Western blot analysis of ferroptosis markers (xCT, GPX4) (**C**) and HSCs activation markers (α-SMA, collagen I) (**D**) in SAT1-overexpressing HSCs with or without spermidine (100 μM) supplementation. **E** Measurements of Fe²⁺, GSH, and MDA levels in the indicated groups. **F** Western blot analysis of GPX4 in SAT1-overexpressing HSCs after treated with cycloheximide (50 μM). (*p < 0.05, **p < 0.01, ***p < 0.001, ****p < 0.0001). Data are representative of three independent experiments.

A paradoxical observation was that while carvedilol decreased xCT and GPX4 protein levels, it increased their mRNA levels (Fig. 6A). This clear discrepancy pointed to a post-transcriptional regulatory mechanism. Since SAT1 catalyzes the N1-acetylation of spermidine, facilitating its export and degradation [21], we hypothesized that SAT1 induces ferroptosis through spermidine depletion in HSCs. High-performance liquid chromatography (HPLC) analysis confirmed that carvedilol treatment led to a profound depletion of intracellular spermidine, an effect that was significantly abrogated by SAT1 knockdown (Fig. 6B). Moreover, supplementation with exogenous spermidine effectively prevented the downregulation of xCT and GPX4 proteins, the accumulation of Fe^2+^ and MDA, and the depletion of GSH in SAT1-overexpressing cells (Fig. 6C, D). Consequently, spermidine also reversed the anti-fibrotic effect (i.e., the loss of activation markers) induced by SAT1 overexpression (Fig. 6E). These data solidify a model where SAT1 drives ferroptosis and HSCs deactivation by depleting spermidine.

To clarify the relationship between intracellular spermidine levels and GPX4 downregulation, we further investigated the stability of the GPX4 protein. The results demonstrated that following exogenous spermidine supplementation, the degradation rate of GPX4 was significantly reduced with a concomitant increase in its stability. This indicates that carvedilol-induced spermidine depletion leads to decreased stability and accelerated degradation of the GPX4 protein, ultimately triggering the onset of ferroptosis (Fig. 6F).

## Discussion

**Fig. 7:**
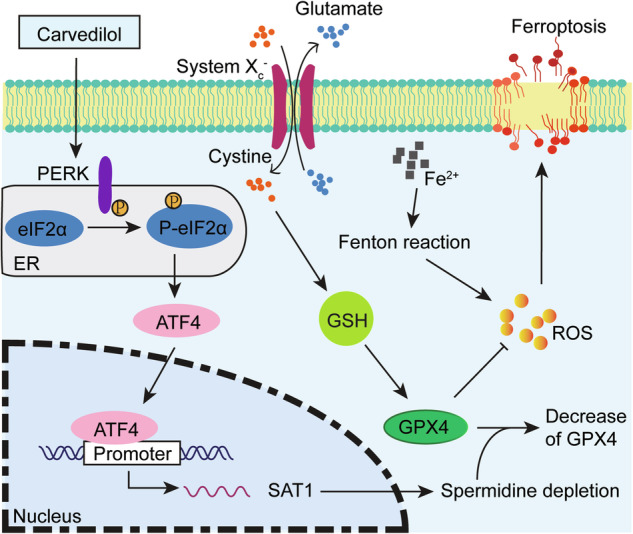
Underlying mechanism of carvedilol triggering HSCs ferroptosis in liver fibrosis via PERK/eIF2α/ATF4 pathway. Carvedilol triggers ER stress in HSCs, activating the PERK/eIF2α/ATF4 signaling axis. ATF4 translocates to the nucleus and binds to the SAT1 promoter, transactivating its expression. Upregulated SAT1 catalyzes the acetylation and depletion of spermidine. Spermidine deficiency leads to the destabilization and degradation of GPX4 protein, ultimately inducing ferroptosis and the subsequent suppression of HSCs activation, thereby ameliorating liver fibrosis.

Liver fibrosis remains a significant therapeutic challenge in hepatology, with current anti-fibrotic strategies often yielding suboptimal outcomes. The pursuit of novel treatments that precisely target the fundamental cellular drivers of fibrosis, particularly HSCs, is therefore of paramount importance. Previous studies have demonstrated that the inactivation of HSCs can effectively improve liver fibrosis [22, 23], but the specific mechanism of action is complex. In this study, we demonstrate a previously unrecognized mechanism underpinning the anti-fibrotic effect of carvedilol, repositioning it from a hemodynamic modulator to a direct inducer of HSCs ferroptosis. We show that carvedilol exerts its anti-fibrotic effects by inducing ferroptosis in HSCs via the PERK/eIF2α/ATF4/SAT1 pathway and subsequent spermidine depletion (Fig. 7). Our findings not only reposition carvedilol as a direct modulator of HSCs activation but also identify the spermidine depletion as a crucial regulatory node in HSCs ferroptosis, offering new insights into the molecular mechanisms governing cell death in fibrotic diseases.

Ferroptosis is a novel type of regulated cell death, and it is implicated in metabolism and redox signaling as well as diverse pathophysiological conditions [24, 25]. However, the role of ferroptosis in liver fibrosis is complex and context-dependent. While its induction in HSCs has emerged as a promising therapeutic strategy [11, 12, 26], its consequences can vary based on the cell type and specific triggers [27, 28]. Our discovery that carvedilol induced ferroptosis in HSCs originated from an unbiased transcriptomic analysis, which was subsequently validated by a collection of definitive phenotypic evidence, including the visualization of characteristic shrunken mitochondria with condensed membranes and loss of cristae via TEM, the biochemical confirmation of ferrous iron and MDA accumulation alongside GSH depletion, and the complete reversal of these effects by the specific ferroptosis inhibitor ferrostatin-1. The consistency of these results across both rat and human HSC lines significantly enhances the translational relevance of our findings, suggesting that ferroptosis induction represents the primary mechanism through which carvedilol compromises HSCs viability and abrogates their pro-fibrogenic phenotype.

A pivotal finding of our study is the identification of SAT1 as a critical mediator of carvedilol’s effect. Although SAT1 has been implicated in ferroptosis across various contexts [29–33], our RNA-seq data revealed SAT1 as one of the most markedly upregulated ferroptosis-related genes following carvedilol treatment, prompting further investigation into its functional role. Subsequent loss- and gain-of-function studies provided compelling evidence for the essential role of SAT1 in this process. These complementary experiments establish SAT1 not merely as a passive biomarker but as an active central executor in this cell death pathway, offering a novel therapeutic target for fibrosis intervention. The ability of SAT1 overexpression to mimic the effects of carvedilol suggests that targeted activation of this enzyme or its regulatory pathways could represent an alternative therapeutic strategy independent of carvedilol administration, potentially enabling the development of more specific anti-fibrotic agents with improved safety profiles.

Mechanistic studies revealed that carvedilol regulates SAT1 expression via an ER stress-ATF4 axis. Transmission electron microscopy first observed dilated ER structures in treated cells, prompting exploration of ER stress pathways. While SAT1 was previously linked to the p53 pathway [34, 35], emerging evidence connects ER stress to ferroptosis [36]. Our data show carvedilol triggers PERK-mediated eIF2α phosphorylation, upregulating ATF4 translation. ChIP-qPCR confirmed that ATF4 directly binds the SAT1 promoter, and ER stress inhibition with ISRIB abrogated SAT1 upregulation. This ER stress-ATF4-SAT1 axis uncovers a novel link between cellular stress responses and ferroptosis, with implications for liver fibrosis and other pathologies. These findings expand understanding of carvedilol’s pleiotropic pharmacological actions in regulating fundamental cellular processes.

A mechanistically insightful observation was the paradoxical increase in *SLC7A11* (xCT) and *GPX4* mRNA levels concurrent with a decrease in their protein levels following carvedilol treatment. This clear discrepancy between transcriptional and translational effects, which contrasts with the results of previous studies on SAT1 and ferroptosis [37, 38], pointed unequivocally to post-transcriptional regulation mechanisms. Given SAT1’s established role in polyamine catabolism, we hypothesized that it might mediate ferroptosis through spermidine depletion. Our HPLC analysis confirmed profound carvedilol-induced spermidine depletion in a SAT1-dependent manner, providing direct metabolic evidence for this hypothesis. The definitive functional evidence came from rescue experiments that exogenous spermidine supplementation in SAT1-overexpressing cells prevented GPX4 protein loss, attenuated toxic metabolite accumulation, and abrogated both ferroptosis execution and HSCs deactivation. These findings establish a definitive causal relationship between SAT1-mediated spermidine depletion and ferroptosis, revealing a previously underappreciated metabolic checkpoint controlling ferroptosis execution in HSCs. This spermidine regulatory axis represents a crucial metabolic vulnerability that could be exploited for therapeutic purposes in fibrotic diseases and possibly other conditions where controlled induction of ferroptosis might be beneficial.

Carvedilol is a well-recognized antioxidant that has been shown to exert anti-ferroptosis effects via attenuating oxidative stress in previous studies [39–41]. However, the biological effects of drugs exhibit remarkable cell type and pathological context dependence, meaning that the same drug may exert distinct effects on different cell types under varying pathological conditions [42]. In the microenvironment of chronic liver disease, activated HSCs are exposed to an inflammatory milieu and possess a redox homeostasis that is fundamentally distinct from that of normal cells, which may represent a critical pathological basis for carvedilol to promote ferroptosis in activated HSCs [1, 43]. Furthermore, the present study found that the mRNA levels of GPX4 and other key factors were elevated in HSCs upon carvedilol treatment, which is not contradictory to the previous finding that carvedilol upregulates GPX4 expression [39]. In contrast to the direct antioxidant activity of carvedilol reported in previous studies, our findings demonstrated that carvedilol modulates spermidine metabolism significantly, leading to an increase in GPX4 consumption. This process specifically disrupts the redox balance and metabolic homeostasis that are already in a precarious state in activated HSCs, ultimately converting this classic antioxidant molecule into a context-dependent ferroptosis modulator [44, 45]. These findings do not contradict the well-established antioxidant properties of carvedilol, but rather point out the pleiotropic role of this drug. More importantly, it uncovers a novel anti-fibrotic strategy that targets the unique metabolic vulnerability of activated HSCs.

Our study, while illuminating this novel pathway, has certain limitations that warrant consideration. First, although we attribute the downregulation of GPX4 and xCT proteins to post-transcriptional mechanisms, the precise degradation pathways (e.g., lysosomal/autophagic degradation or oxidative modification) remain to be fully characterized. Second, our findings are primarily derived from a CCl_4_-induced fibrosis model, primarily reflecting chemical injury, and the generalizability of our findings to other fibrotic etiologies (e.g., non-alcoholic steatohepatitis or viral hepatitis) merits further investigation. Finally, while SAT1 was identified as a key mediator, the upstream signaling events linking carvedilol to ER stress initiation require more detailed elucidation. These limitations, however, highlight fertile ground for future research to validate SAT1 enzymatic function, explore in vivo ferroptosis markers, dissect alternative protein degradation pathways, and evaluate the mechanism across diverse fibrotic models.

In conclusion, our work provides a comprehensive mechanistic framework for the direct anti-fibrotic action of carvedilol. We have identified a linear pathway from carvedilol-induced ER stress to HSCs ferroptosis via the ATF4/SAT1/spermidine depletion axis. These findings not only expand our understanding of carvedilol’s pharmacology but also unveil novel therapeutic targets, most notably SAT1 and spermidine metabolism, for the treatment of fibrotic liver diseases. Future studies should explore the universality of this mechanism in other organ fibrosis models, investigate potential biomarkers for patient stratification, and examine the therapeutic potential of more specific modulators targeting this newly identified pathway.

## Materials and methods

### Animal experiments

Male C57BL/6 mice (6 weeks old) were purchased from Pengyue Biotechnology Corporation, Ltd. (Jinan, Shandong, China). The animals were housed under standard conditions at the Animal Center of Shandong Provincial Hospital. After one week of adaptation, a liver fibrosis model was established via intraperitoneal injection of CCl_4_ (1 ml/kg body weight, 1:4 v/v in olive oil) twice a week. Control mice received an equal volume of olive oil. After 2 weeks, mice were randomly grouped and administrated either carvedilol (10 mg/kg) or vehicle (water) daily by oral gavage for 6 weeks. After the last administration, all mice were euthanized under anesthesia (by intraperitoneal injection of 50 mg/kg of pentobarbital sodium), and then blood and liver tissues were collected for subsequent analysis.

### Cell culture and treatment

The rat HSC line (HSC-T6) and human HSC line (LX-2) were purchased from Procell (Wuhan, China). Cells were cultured in complete Dulbecco’s modified essential medium (DMEM, #11995065, Gibco) supplemented with 10% fetal bovine serum (FBS, #A5256701, Gibco) and 1% penicillin/streptomycin (#15640055, Gibco). Carvedilol (#S1831, Selleck) and Ferrostatin-1(#HY-100579, MedChemExpress) were dissolved in dimethyl sulfoxide (DMSO; #D8371, Solarbio) according to the specification. Spermidine (HY-B1776, MedChemExpress) was dissolved in sterile water. Control groups received an equal volume of DMSO, with the final concentration of DMSO not exceeding 0.1% in all experiments.

### Lentivirus transfection

For gene knockdown, short hairpin RNAs (shRNAs) targeting SAT1 or non-targeting control shRNAs were cloned into the GV493 lentiviral vector (Shanghai Genomics Chemical Co., Ltd.). For SAT1 overexpression, the coding sequence was inserted into the vector plasmid CV374 (Shanghai Genomics Chemical Co., Ltd.), and this vector is equipped with a tetracycline-inducible switch system to achieve regulated gene expression. Stable cell lines were established following lentiviral infection and puromycin selection. The target interfering sequences are represented in the Supplement Table 1.

### Cell viability analysis

Cell viability was evaluated using the Cell Counting Kit-8 (CCK-8, #A311-01, Vazyme) according to the manufacturer’s instructions. Cells were cultured in 96-well plates overnight, and then were treated with different reagents for 24 h. After that, 10 μl CCK-8 reagents were added to each well and incubated at 37°C in 5% CO_2_ for 1 h, and the optical density (OD) values were measured at 450 nm.

### Western blotting

The total proteins of cells and liver tissue were extracted by RIPA with PMSF (#R0020, Solarbio). Protein concentrations were measured using a BCA protein assay kit (#P0012S, Beyotime). Samples were subjected to SDS-PAGE gels (#PG212, YamayBio) and transferred to PVDF membranes (#IPVH00010, Millipore) and blocked with TBST (#PS103S, YamayBio) and 5% milk for 1 h. The membranes were incubated with the primary antibody diluted in primary antibody dilution buffer (#PS114, YamayBio) overnight at 4 °C. The primary antibodies used are listed in the Suppl. Table 2. Then the membranes were incubated with secondary anti-rabbit antibody (1:5000, #ab288151, # Abcam) or anti-mouse antibody (1:5000, #ab6789, Abcam) for 1 h at room temperature and were visualized using an ECL kit (#WBKLS0100, Millipore).

### ROS assay

Intracellular ROS was assayed with the Reactive Oxygen Species Assay Kit (#CA1410, Solarbio). Cells were seeded in 24-well plates and pretreated with relevant reagents for 24 h. The diluted DCFH-DA probe (10 μM) was added to each well, and the cells were incubated in an incubator at 37 °C for 20 min. Then the cells were washed with serum-free cell culture medium, and the ROS levels were determined with fluorescence microscopy.

### Iron assay

Intracellular iron was assayed with FerroOrange (#HY-D1913, MedChemExpress) and an iron assay kit (#E1046, Applygen Technologies Inc.). Cells were seeded in 96-well plates and pretreated with relevant reagents for 24 h. After the diluted FerroOrange probe (1 μM) was added, the cells were incubated in an incubator at 37 °C for 30 min. As for the iron assay kit, cells were gently collected in PBS solution (#BL601A, Biosharp) with a cell spatula and detected according to the manufacturer’s instructions.

### GSH/MDA assay

After being treated with relevant reagents, cells were gently collected in PBS solution. The GSH and MDA concentrations in cell lysates were assessed using the reduced glutathione content assay kit (#BC1175, Solarbio) and the malondialdehyde detection kit (#E2019, Applygen Technologies Inc.), according to the manufacturer’s instructions.

### Histopathological examination, immunohistochemical staining and blood analysis

Liver tissues were fixed in tissue fixation fluid for 24 h and embedded in paraffin blocks. Then, tissue slides of serial 5-μm-thick sections were stained with hematoxylin and eosin (H&E), Sirius Red and Masson for histological analysis. According to the manufacturer’s instructions for DAB chromogenic reagent, some slides were incubated with the appropriate primary antibodies. The secondary anti-rabbit antibody (#ab150075, Abcam) or anti-mouse antibody (#ab150113, Abcam) was purchased from Abcam for fluorescence. Alanine aminotransferase (ALT) and aspartate aminotransferase (AST) levels in the mouse serum were measured using an automatic biochemical analyzer (Chemray 800; Rayto) according to the instructions.

### Immunofluorescence staining

Cells were seeded in confocal dishes and treated. Then, cells were fixed in 4% paraformaldehyde for 15 min and incubated with immunofluorescence blocking solution for 60 min at room temperature. Cells were incubated with primary antibodies overnight at 4 °C and were incubated with secondary antibodies for 1 h at room temperature. Fluorescence was visualized with a NIKON ECLIPSE Ti confocal microscope (Nikon, Japan).

Quantitative real-time polymerase chain reaction (RT-qPCR) and PCR array sequencing of RNA (RNA-seq)

Total RNA was extracted from cells utilizing RNA extraction reagent (#AG21101, Accurate Biology), and a reverse transcription kit (#AG11728, Accurate Biology) was used to synthesize cDNA. A SYBR Green PCR kit (#AG11701, Accurate Biology) was used for RT-qPCR. The mRNA levels were normalized to those of GAPDH. The primers used are represented in Supplement Table 3. RNA-seq of T6 cells was performed by Lianchuan Biotechnology Co., Ltd.

### Chromatin immunoprecipitation (ChIP) assay

Cells were seeded in 10 cm culture dishes (1 × 10⁷–5 × 10⁷ cells per dish) and treated accordingly for 24 h. The cells were fixed with 0.1% formaldehyde to cross-link proteins to DNA, and the cross-linking reaction was quenched by adding glycine to a final concentration of 125 mM. The cells were washed with PBS and collected using a cell scraper. Chromatin was fragmented using a sonicator, and a portion of the sample was reverse-cross-linked and electrophoresed to verify fragment size distribution. After quantifying the chromatin concentration, a certain amount of chromatin was immunoprecipitated by incubating with protein A/G agarose beads (#G2210-50T, Servicebio) and antibody. An appropriate isotype IgG was used as a negative control. The immunocomplexes were washed and then reverse-cross-linked, followed by DNA extraction and purification. The resulting DNA was subjected to RT-qPCR analysis to examine the enrichment of specific genomic regions.

### Transmission electron microscopy (TEM)

After being treated with relevant reagents for 24 h, we fixed cells with 2% glutaraldehyde and 1%osmiumtetroxide and performed graded dehydration. Subsequently, we made ultrathin and stained slices with 1% lead citrate and 10% uranyl acetate. Images were obtained through a transmission electron microscope (HT7800, Hitachi).

### Cycloheximide chase assay

Cycloheximide (CHX) was used to inhibit protein synthesis. Cells were treated with CHX (50 μM, HY-12320, MCE) for 0, 2, 4, and 8 h, and lysed for Western blot analyses as described previously.

### Statistical analysis

Statistical analyses were performed using GraphPad Prism 10 (GraphPad Software). Data represent biological replicate (*n*), and are depicted in figures as mean values ± standard deviations (SDs). For samples with a normal distribution, means were compared by Student’s *t* test (two samples) or ANOVA ( > 2 samples) followed by Tukey’s post hoc analysis. Nonparametric tests (Kruskal−Wallis) followed by Dunn’s multiple comparisons were used otherwise. Differences were considered statistically significant at *p* < 0.05.

## Supplementary information

Supplementary Tables and Figures

Original Western Blot

aj-checklist

## Data Availability

The RNA-seq datasets generated and analysed during the current study are available in the Sequence Read Archive repository (PRJNA1354504). The other data presented in this article will be made available by the corresponding author upon request.
