# Supplementary material for: Carvedilol triggers ferroptosis in hepatic stellate cells via the ATF4/SAT1 axis promoting spermidine depletion to ameliorate liver fibrosis
Source: Cell Death Dis. 2026 May 22;17(1):641. doi: 10.1038/s41419-026-08898-5 (PMC13369927; doi:10.1038/s41419-026-08898-5)
Supplement: Supplementary file 1 — Supplementary Tables and Figures [file 41419_2026_8898_MOESM1_ESM.pdf]

Supplementary Table 1 Target sequences for RNAi.

Species	Gene	Target sequence (5′-3′)
Rat	Sat1-RNAi-1	GCTGCAGCAGTATGCACTTCT
Rat	Sat1-RNAi-2	ACCTATGACCCATGGATTGGC
Rat	Sat1-RNAi-3	GCAGAGTGGAATGAACCATCT
Human	SAT1-RNAi-1	GCAGCATGCACTTCTTGGTAG
Human	SAT1-RNAi-2	GGAATGAACCATCCATCAACT
Human	SAT1-RNAi-3	GGGTTGGAGACTGTTCAAGAT

Supplementary Table 2 Information Table of Antibodies Used in Experiments

Antibody Name	Dilution Ratio	Catalog Number	Brand
Collagen I (COL1A1)	1:1000	#ab260043	Abcam
Alpha Smooth Muscle Actin (α-SMA)	1:1000	#14395-1-AP	Proteintech
Transferrin Receptor (TRFC)	1:1000	#10084-2-AP	Proteintech
xCT	1:1000	#DF12509	Affinity Biosciences
Glutathione Peroxidase 4 (GPX4)	1:1000	#DF6701	Affinity Biosciences
Spermidine/Spermine N(1)-Acetyltransferase 1 (SAT1)	1:500	#10708-1-AP	Proteintech
PERK	1:1000	#24390-1-AP	Proteintech
eIF2α	1:1000	#11170-1-AP	Proteintech

Phospho-eIF2α	1:1000	#28740-1-AP	Proteintech
ATF4	1:1000	#10835-1-AP	Proteintech
P62	1:1000	#5114T	Cell Signaling Technology
LC3B	1:1000	#ab192890	Abcam
Bcl-2	1:1000	#ab182858	Abcam
Bax	1:1000	#50599-2-Ig	Proteintech
Cleaved Caspase-3	1:1000	#25125-1-AP	Proteintech
Beta-Actin (β-Actin)	1:1000	#20536-1-AP	Proteintech
GAPDH	1:1000	#AF7021	Affinity Biosciences

Supplementary Table 3 Primers for RT-qPCR.

Species	Primer	Forward	Reverse
Rat	Collagen I	CACTGCAAGAACAGCGTAGC	AAGTTCCGGTGTGACTCGTG
Rat	α-SMA	GGGAGTGATGGTTGGAATGGG	CCGTTAGCAAGGTCGGATGC
Rat	xCT	ATCTTCGATACAAACGCCCAGAT	AAAGGGAGAGGACAACCATGAAG
Rat	GPX4	GGATGAAAGTCCAGCCCAAGG	CGCAGCCGTTCTTATCAATGAG
Rat	PERK	GAGTCCGATGACGACGTGGAA	TCGTCCATCTAAAGTGCTGATGA
Rat	eIF2α	AGGACTGCCTGGGTCTTTGA	GCTCGAATCTTGACAGCTTGTG
Rat	ATF4	AGTCTGCCTTCTCCAGGTGTTC	GCTGTCTTGTTTTGCTCCATCTT
Rat	Sat1	ACTGGACCCCTGAAGGACACA	ACCAAAGCCTCGGTAATCACTC
Rat	GAPDH	TGGTGAAGGTCGGTGTGAAC	TTGCCGTGGGTAGAGTCATA

Human	Collagen I	CAGGGCGACAGAGGCATAAAG	GGACCTTGTTCACCAGGAGAG
Human	α-SMA	GGACCTTGTTCACCAGGAGAG	CGGCTTCATCGTATTCCTGTTTG
Human	xCT	CGGTGGTGTGTTTGCTGTCT	GCTGGTAGAGGAGTGTGCTTG
Human	GPX4	TGGCCTTCCCGTGTAACCAG	CCGTTCACGCAGATCTTGCT
Human	PERK	CTGCTTCTACAGCGTACCCA	AAATCCGGCTCTCGTTTCCA
Human	eIF2α	GGCAGGAATGAGTGTGTGGT	TCAGCAACATGACGAAGAATGC
Human	ATF4	GGCCAAGCACTTCAAACCTC	GACTGACCAACCCATCCACA
Human	SAT1	ACTCCGGAAGGACACAGCATT	CTGATCCTATGCCAAAGCCTCT
Human	GAPDH	GTGAAGGTCGGAGTCAACGG	GCAACAATATCCACTTTACCAGAGT

Supplementary Figures

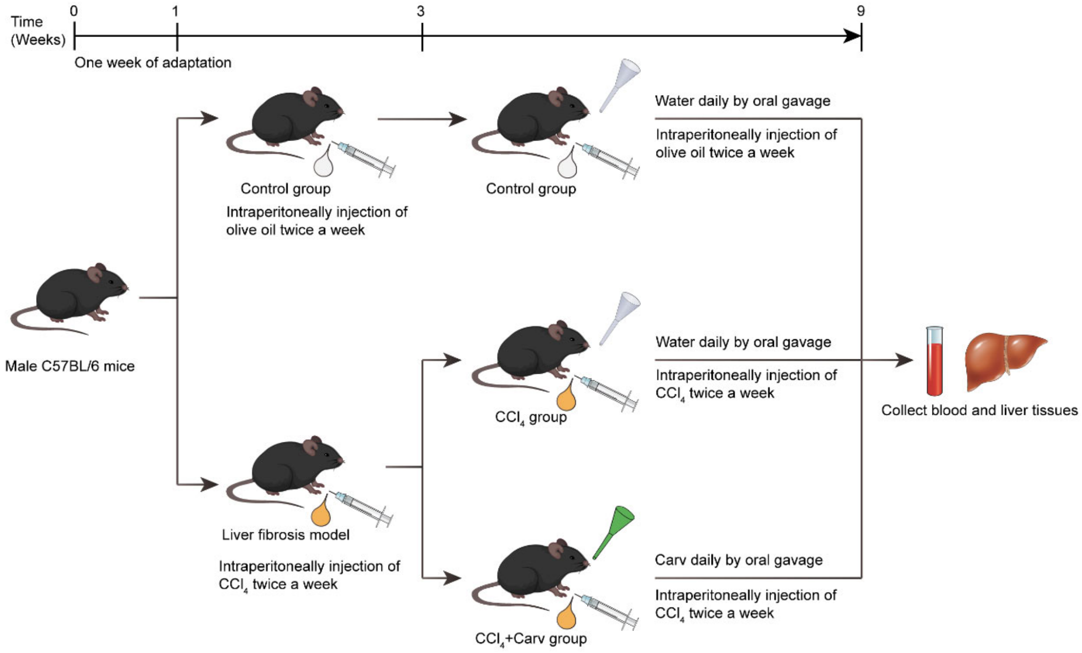

Suppl. Figure 1. Therapeutic potential of carvedilol *in vivo* using a CCl_4-induced mouse model of liver fibrosis.

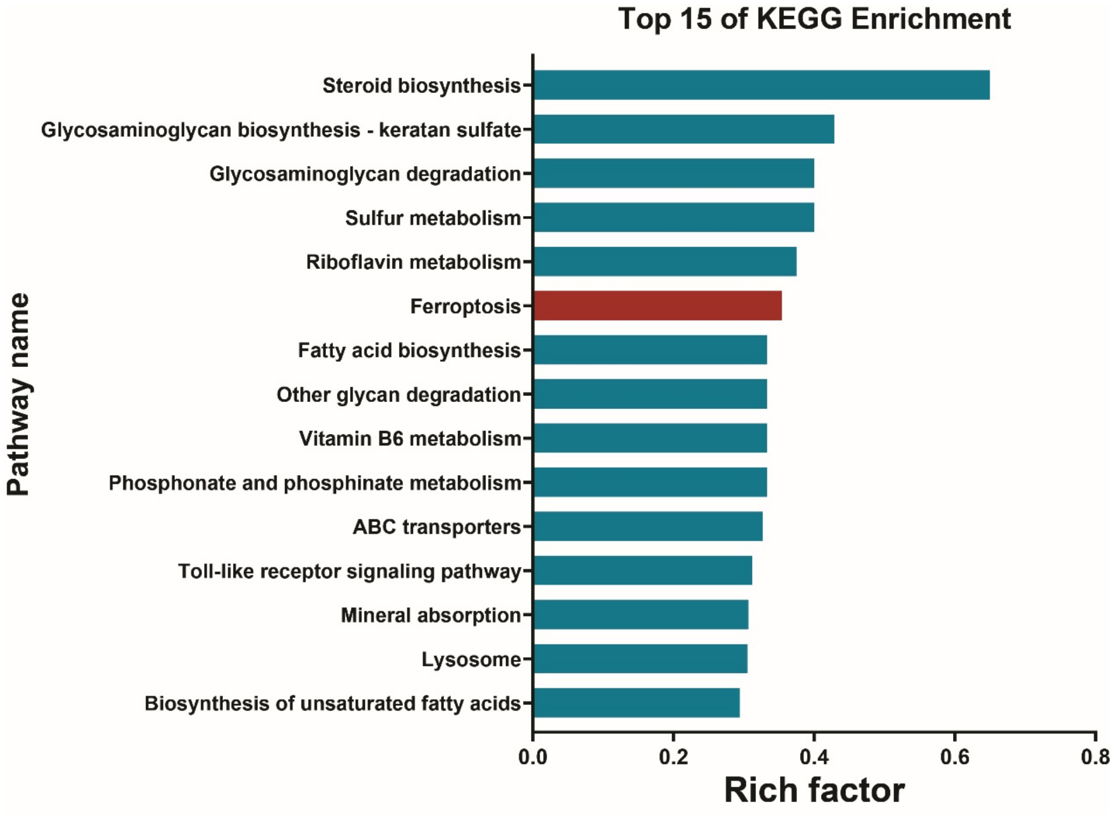

Suppl. Figure 2. Kyoto Encyclopedia of Genes and Genomes (KEGG) pathway enrichment analysis in HSCs treated with 30 μM carvedilol.

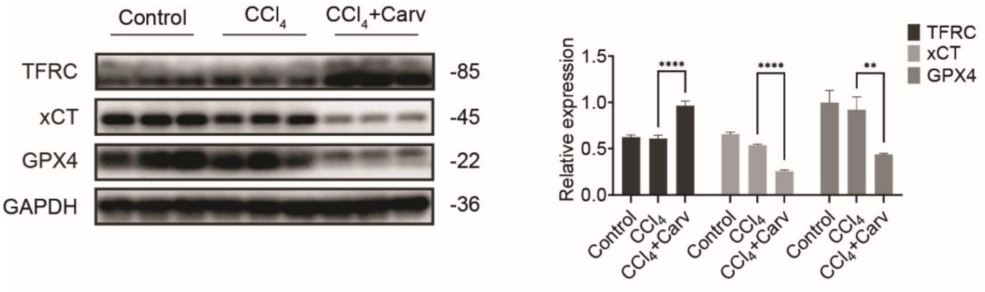

Suppl. Figure 3. Western blot analysis of TFRC, xCT, and GPX4 for CCl_4-induced mouse model of liver fibrosis.

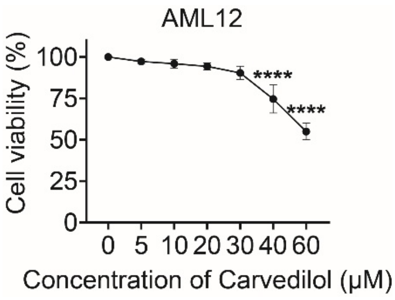

Suppl. Figure 4. Cell viability of AML12 measured by CCK-8 assay after carvedilol treatment.

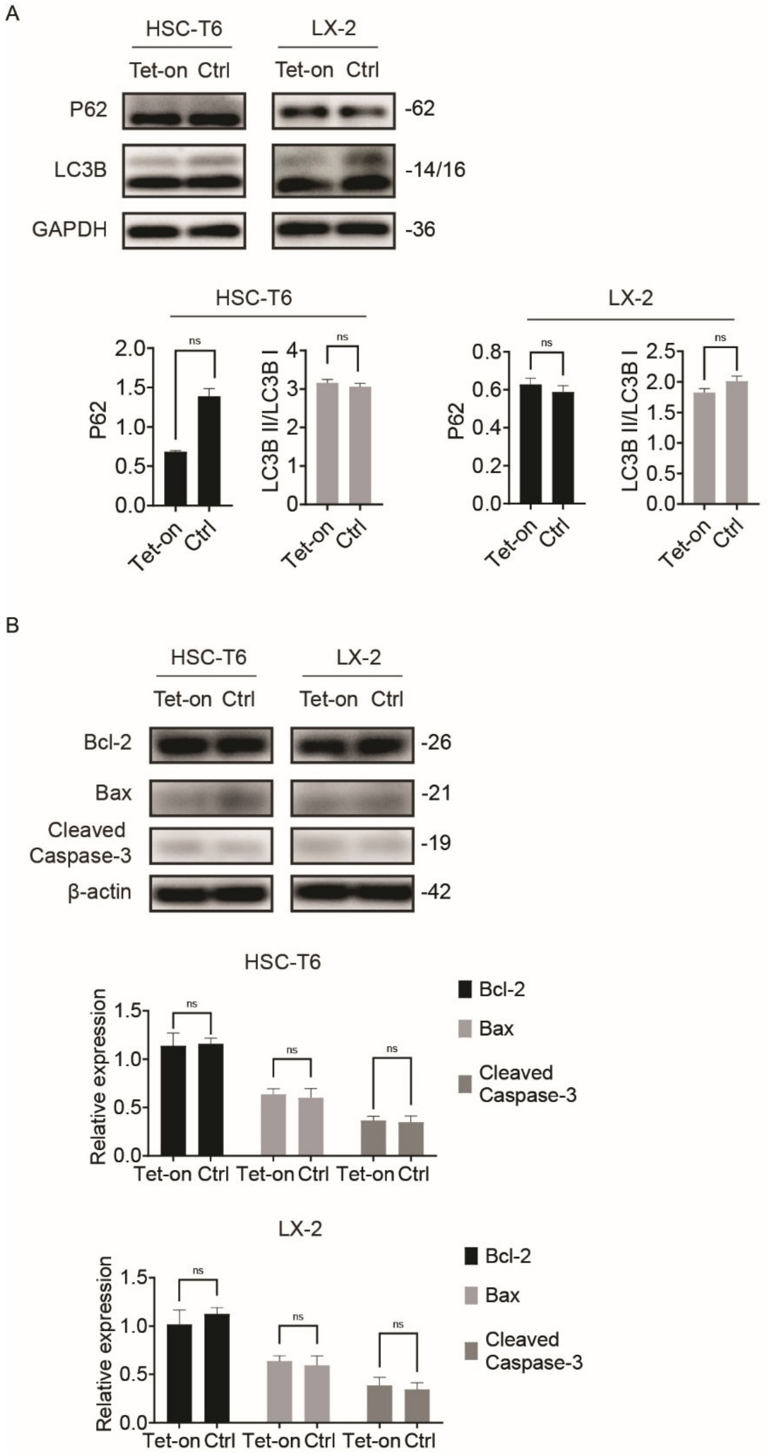

Suppl. Figure 5. Western blot analysis of autophagy (P62, LC3B) (A) apoptotic (Cleaved Caspase-3, Bax, Bcl-2) (B) in SAT1-overexpression HSCs.

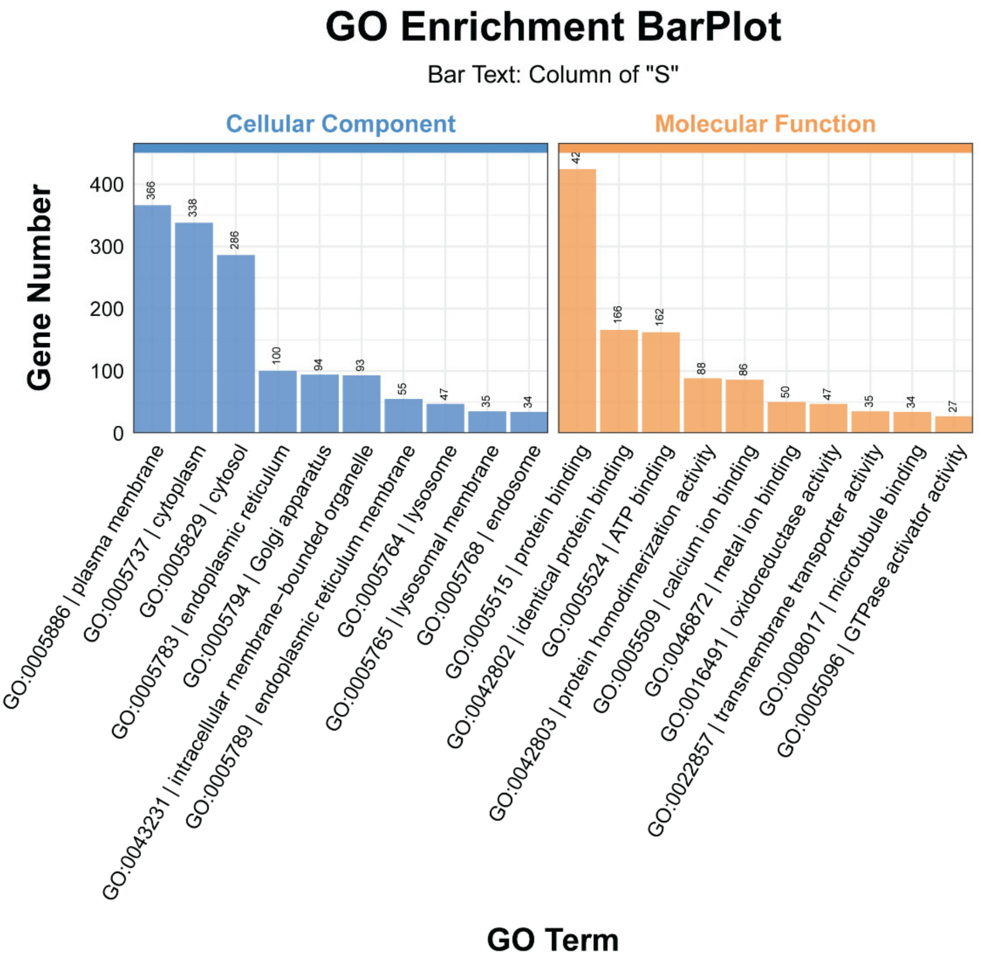

Suppl. Figure 6. Pathway analysis of RNA-seq data suggested ER stress involvement.

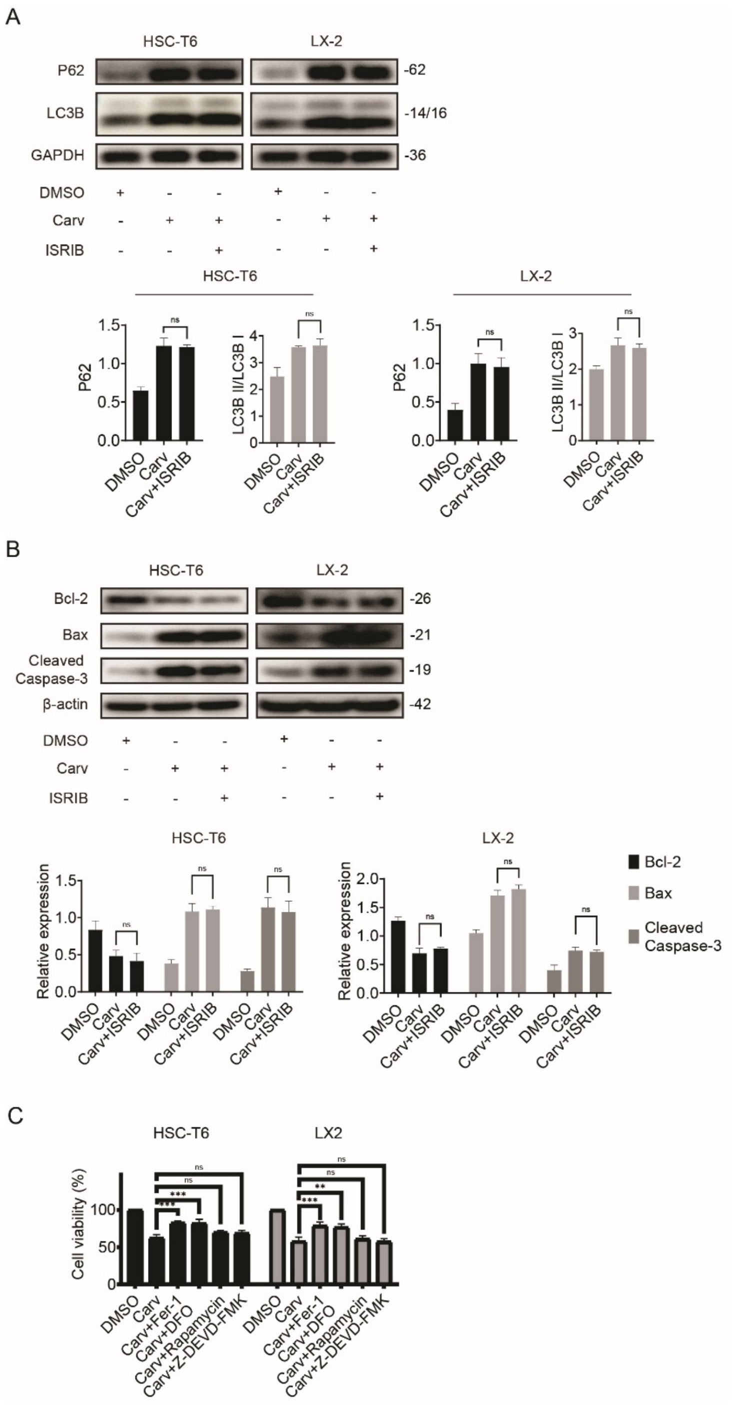

Suppl. Figure 7. Western blot analysis of autophagy (P62, LC3B) (A) apoptotic (Cleaved Caspase-3, Bax, Bcl-2) (B) in HSCs treated with carvedilol (30 μM) and ISRIB (200 nM). (C) Cell viability of HSCs measured by CCK-8 assay after carvedilol, Fer-1 (1 μM), DFO (100 μM), rapamycin (100 nM), and Z-DEVD-FMK (100 nM) treatment.
