# Supplementary material for: Carvedilol triggers ferroptosis in hepatic stellate cells via the ATF4/SAT1 axis promoting spermidine depletion to ameliorate liver fibrosis
Source: Cell Death Dis. 2026 May 22;17(1):641. doi: 10.1038/s41419-026-08898-5 (PMC13369927; doi:10.1038/s41419-026-08898-5)

Original Western Blot

Figure 1A

HSC-T6

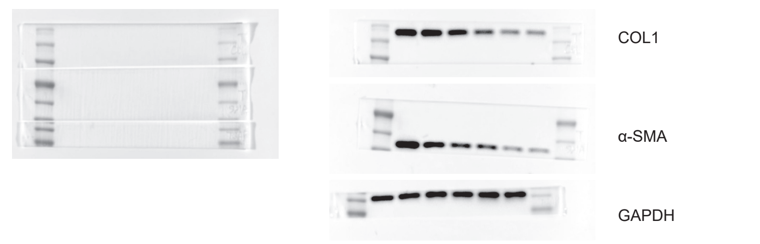

LX-2

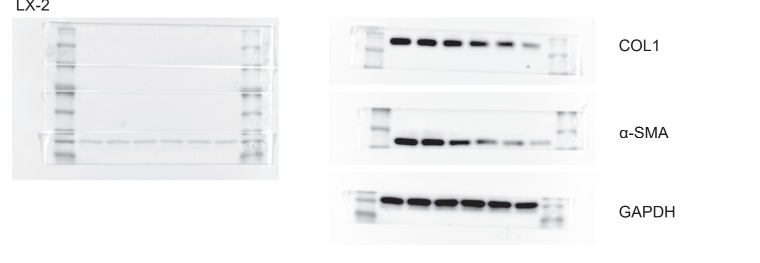

Figure 1E

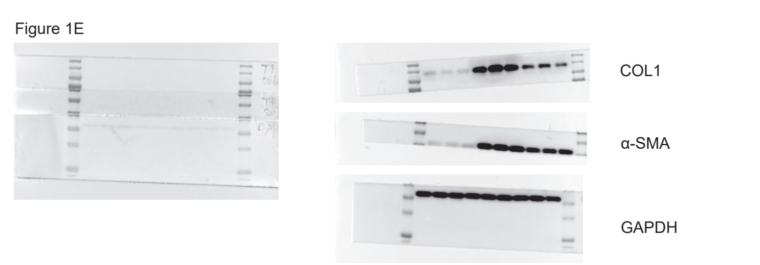

Figure 2C

HSC-T6

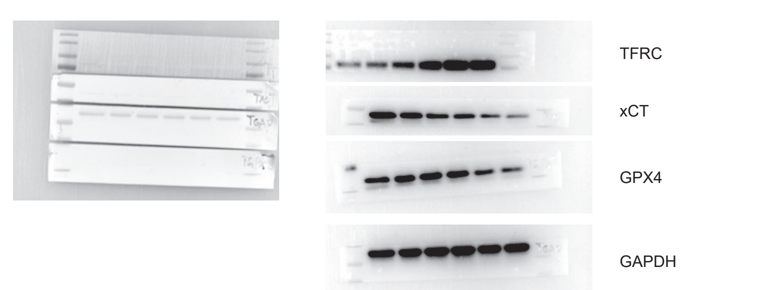

LX-2

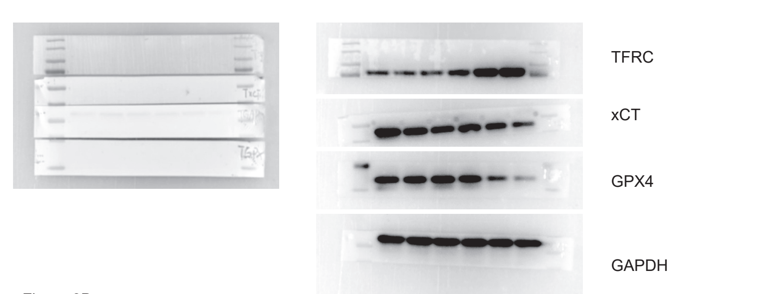

Figure 3B

HSC-T6

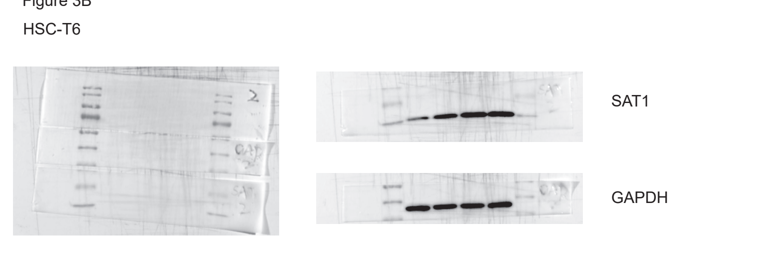

LX-2

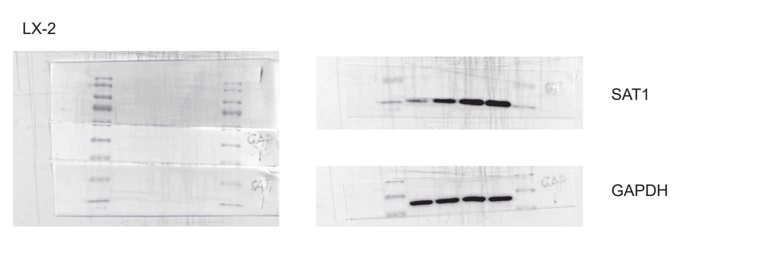

Figure 3F

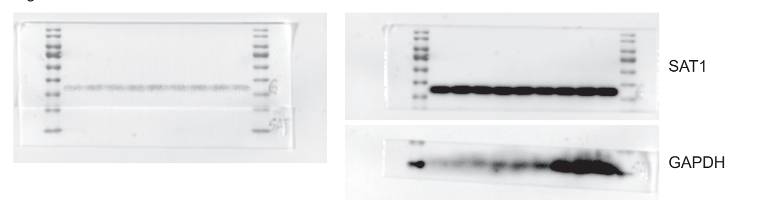

Figure 4A

HSC-T6

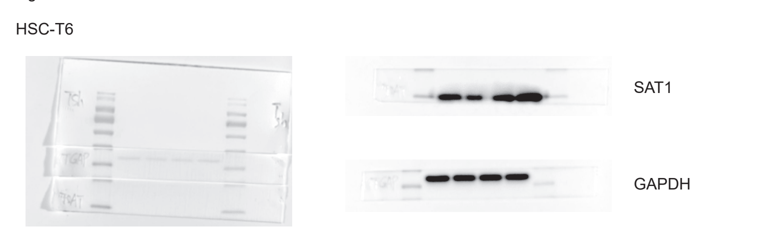

LX-2

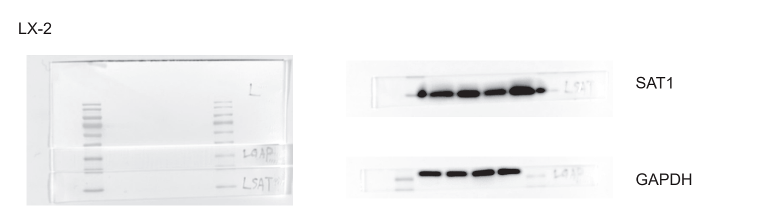

Figure 4B

HSC-T6

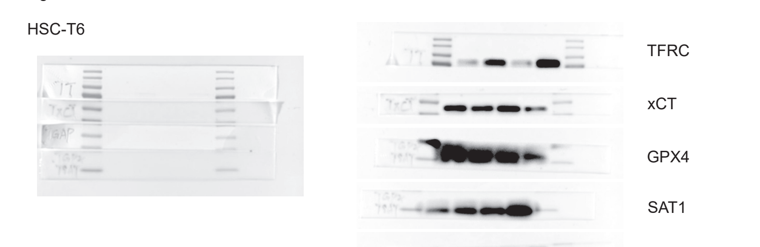

LX-2

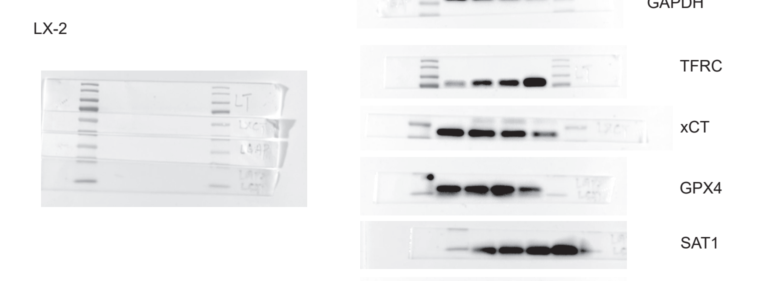

Figure 4C

HSC-T6

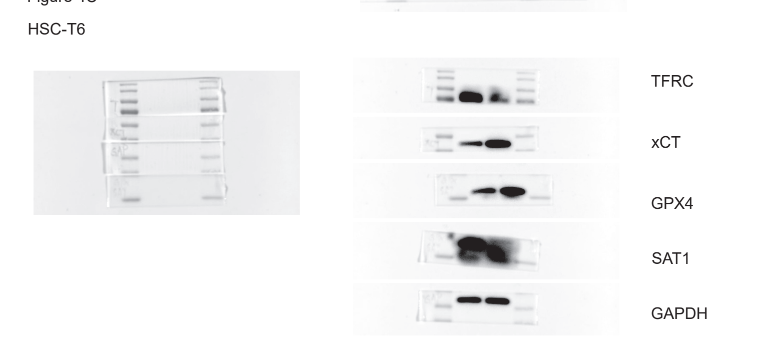

LX-2

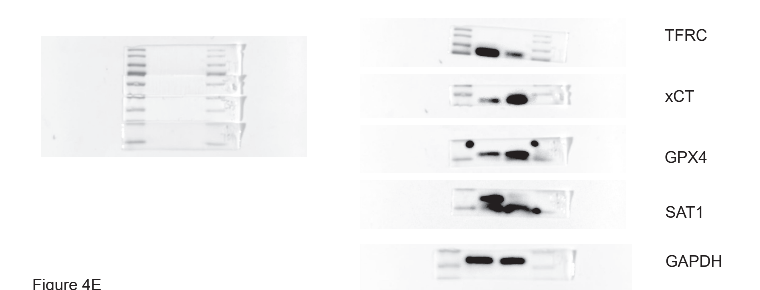

Figure 4E

HSC-T6

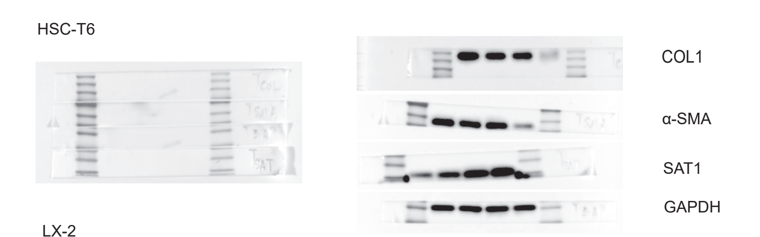

LX-2

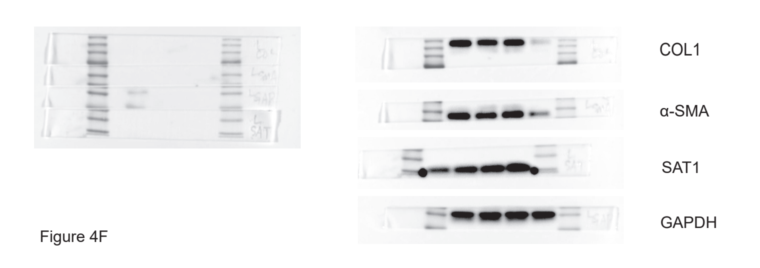

Figure 4F

HSC-T6

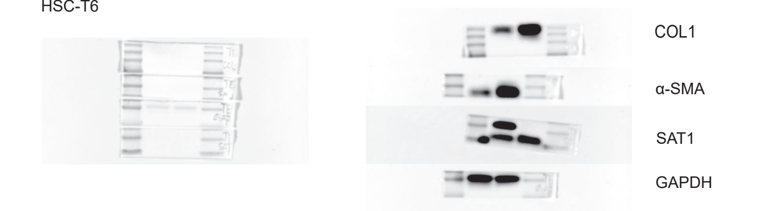

LX-2

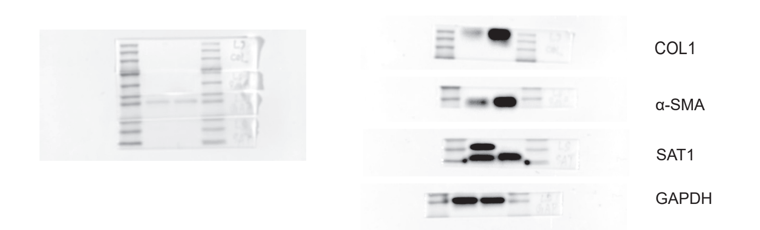

Figure 5B

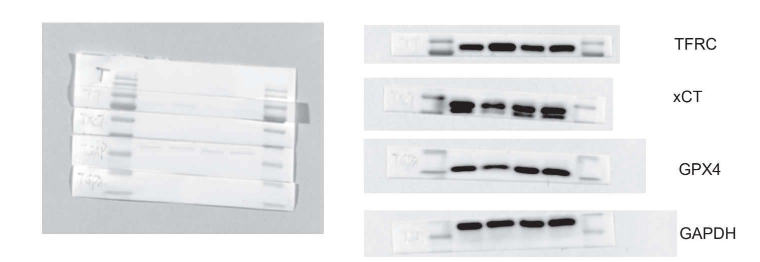

Figure 5C

HSC-T6

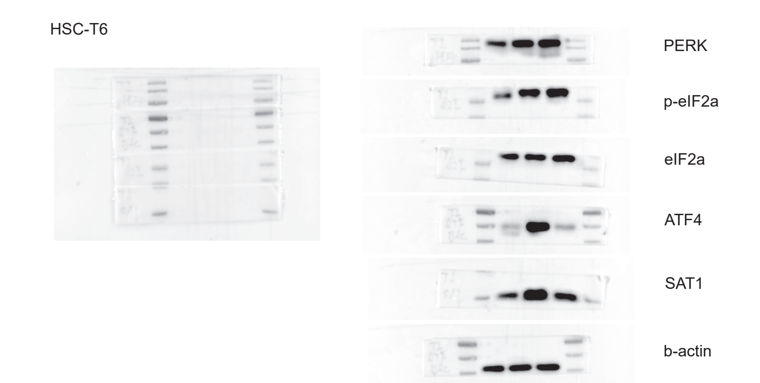

LX-2

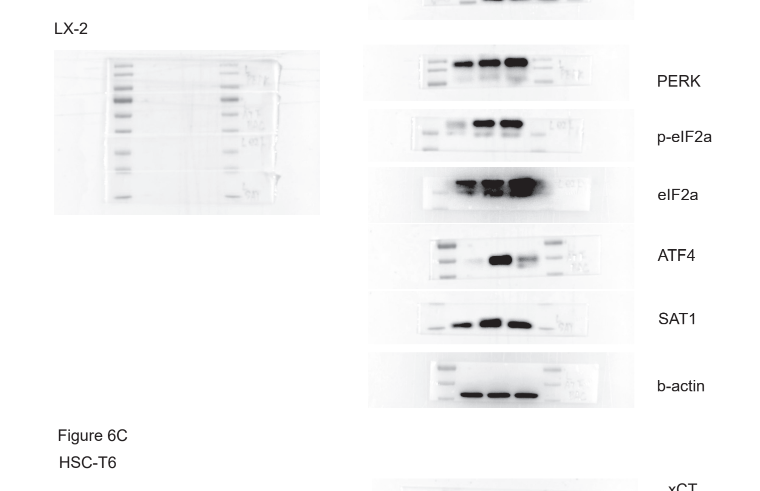

Figure 6C

HSC-T6

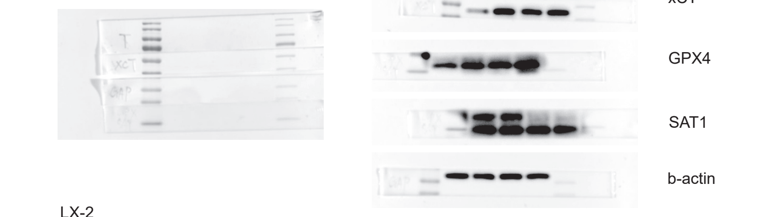

LX-2

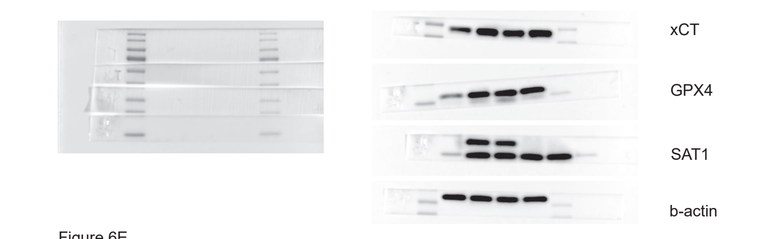

Figure 6E

HSC-T6

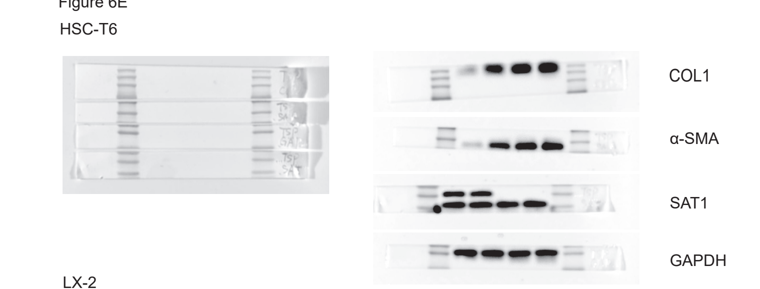

LX-2

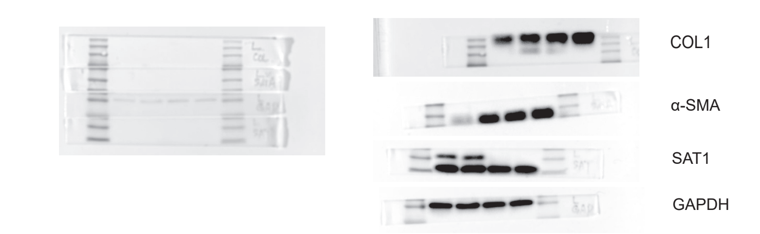

Figure 6F

HSC-T6

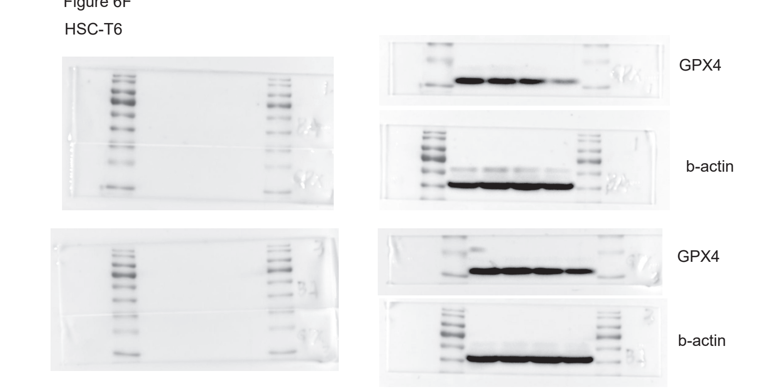

LX-2

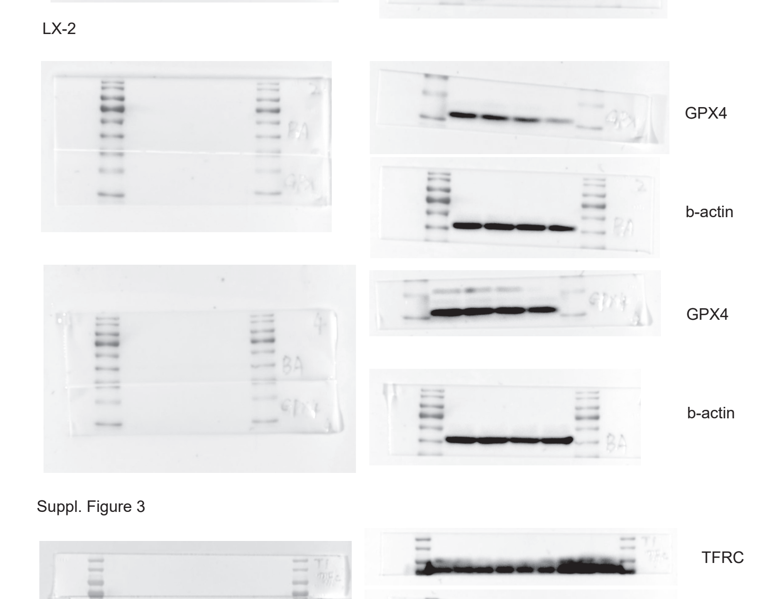

Suppl. Figure 3

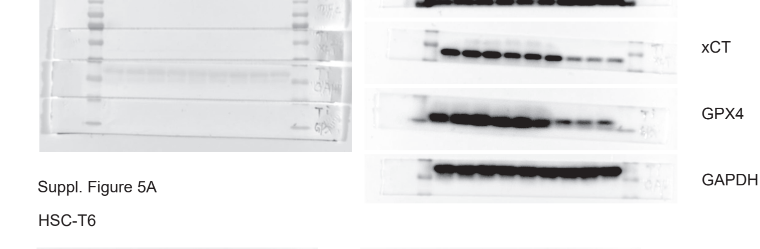

Suppl. Figure 5A

HSC-T6

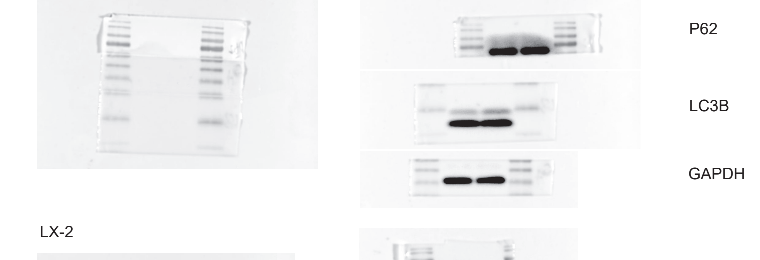

LX-2

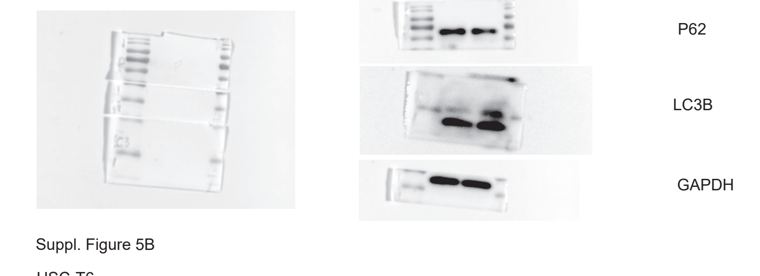

Suppl. Figure 5B

HSC-T6

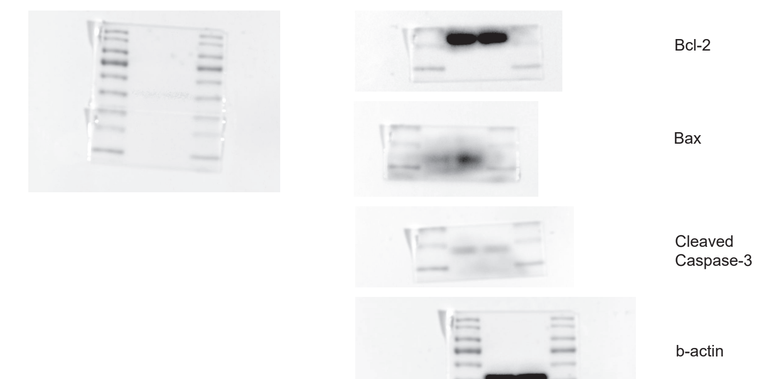

LX-2

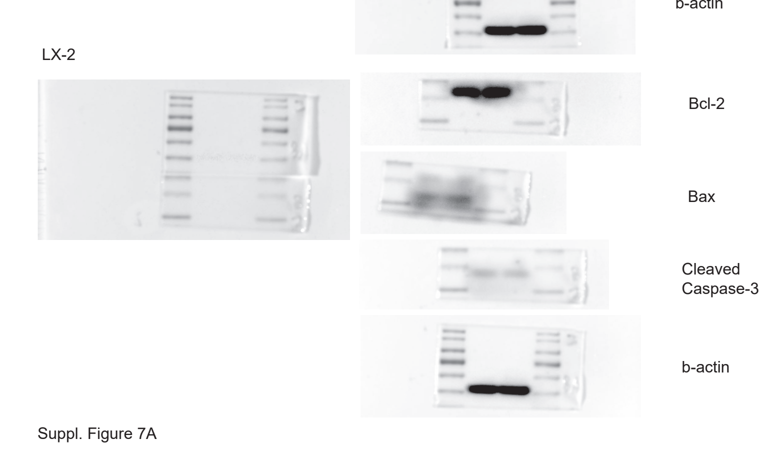

Suppl. Figure 7A

HSC-T6

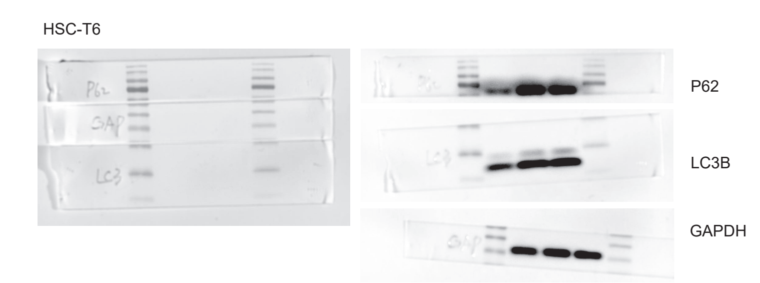

LX-2

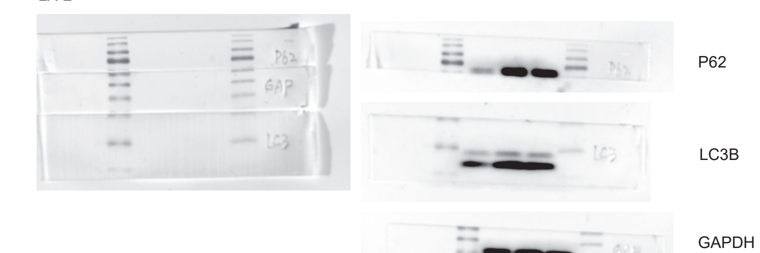

Suppl. Figure 7B

HSC-T6

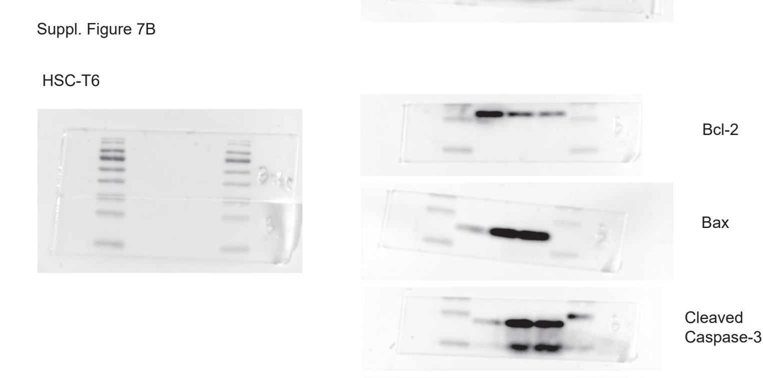

LX-2

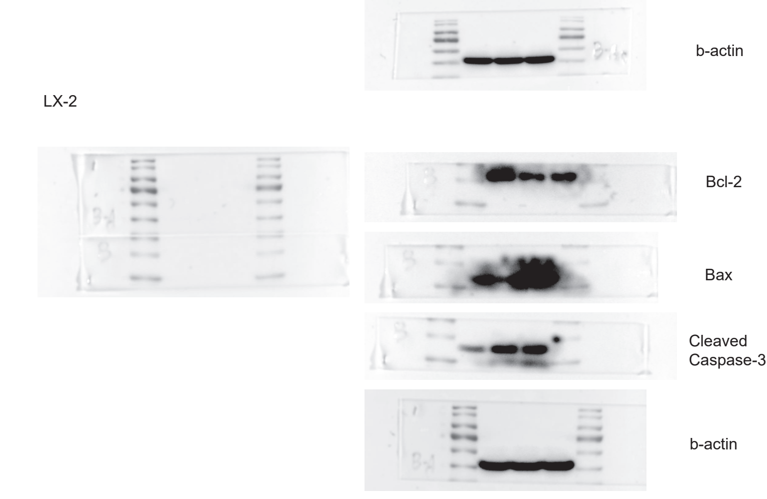

Supplement: Supplementary file 2 — Original Western Blot [file 41419_2026_8898_MOESM2_ESM.pdf]
